# Identification and lipolytic activity of yeasts isolated from foods and wastes

**DOI:** 10.1080/21501203.2020.1745922

**Published:** 2020-03-26

**Authors:** Nattakorn Kuncharoen, Sujitra Techo, Ancharida Savarajara, Somboon Tanasupawat

**Affiliations:** a Department of Biochemistry and Microbiology, Faculty of Pharmaceutical Sciences, Chulalongkorn University, Bangkok, Thailand; b Mahidol University, Nakhonsawan Campus, Nakhonsawan, Thailand; c Department of Microbiology, Faculty of Science, Chulalongkorn University, Bangkok, Thailand

**Keywords:** Diversity, lipolytic activity, Thai fermented foods, wastes of the palm oil industry

## Abstract

Thirty-three yeasts were isolated from palm oil industrial wastes and traditional fermented foods in Thailand. Based on the analysis of the sequences of the D1/D2 region of the large subunit ribosomal RNA gene (LSU rDNA) and their phenotypic characteristics, they were identified as *Pichia kudriavzevii* (11 isolates), *Candida ethanolica* (1 isolate), *Clavispora lusitaniae* (2 isolates), *Ogataea polymorpha* (1 isolate), *Hanseniaspora opuntiae* (1 isolate), *Lodderomyces elongisporus* (1 isolate), *Saccharomyces cerevisiae* (2 isolates), *C. tropicalis* (5 isolates), *Yarrowia lipolytica* (2 isolates), *Magnusiomyces ingens* (1 isolate), and *Magnusiomyces capitatus* (3 isolates), *Trichosporon insectorum* (1 isolate), and *Trichosporon asteroides* (2 isolates). Seven strains, *T. insectorum* 4E-1D, *M. capitatus* 5E-1T and 5E-2D, *T. asteroides* 8E-1T and 8E-1D, and *Y. lipolytica* Fy-12 and Fy-13, showed high lipolytic activity ranged from 5.21 ± 0.09 to 45.68 ± 2.37 U/mL. Moreover, these seven strains exhibited good lipolytic activity after culturing in the medium containing palm oil (11.79 ± 0.67 to 28.19 ± 4.84 U/mL) and soy oil (9.14 ± 1.08 to 22.97 ± 0.69 U/mL) as lipase inducers. The result of this study suggests that the palm oil industrial wastes and Thai fermented foods could be promised as the invaluable sources of lipolytic yeasts.

## Introduction

Lipases (triacylglycerol hydrolases, E.C. 3.1.1.3) are a class of hydrolase enzymes which can hydrolyse triglycerides at the lipid-water interface to free fatty acids, monoglycerides, diglycerides and glycerol (Fickers et al. 2004; Vitisnat et al. 2013). Moreover, lipases catalyse a wide range of reactions, including hydrolysis, inter-esterification, alcoholysis, acidolysis, aminolysis and transesterification (Wang et al. 2007). Lipases have tremendous potential for contributing to the multibillion-dollar underexploited biological industry such as food, pharmaceutical, biomedical sciences, and cosmetic industry (Pandey et al. 1999).

Lipases have been found in animals, plants and microorganisms. However, enzymes from microbial sources are currently receiving more attention because of their interesting characteristics, such as stability in an organic solvent and high substrate specificity (Salihu et al. 2012). Microorganisms are recognised as sources of extracellular lipases, especially yeasts and filamentous fungi such as *Candida* spp., *Yarrowia lipolytica, Rhodotorula* spp., *Pichia* spp., *Saccharomycopsis crataegensis, Torulospora globosa, Trichosporon asteroides, Mucor* spp. and *Aspergillus* spp. (Sharma et al. 2001; Vakhlu and Kour 2006; Ertugrul et al. 2007). Furthermore, some of the yeast strains are considered as non-pathogenic, the processes for lipase production based on yeasts have been classified as GRAS (generally regarded as safe) (Wang et al. 2007).

Naturally, yeasts and moulds have ability to produce lipases are found in several habitats including waste of vegetable oil, dairy product industries, seeds, and deteriorated food (Singh and Mukhopadhyay 2012). The present study aimed to isolate and analyse the diversity of lipolytic yeasts from foods and wastes in Thailand. We also carried out the hydrolysis of various substrates by crude lipases produced by the selected isolates. To our knowledge, this is the first report on the lipolytic yeasts associated with fermented foods and the palm oil industrial wastes in Thailand.

## Materials and methods

### Isolation of lipolytic yeasts

The palm oil industrial wastes including palm cake, palm shell and palm fibre were collected from 2 factories, Eastern Palm Oil Co., Ltd. and Suksomboon Co., Ltd. in Chonburi province and fermented food samples: fermented beef (*Mam-Neua*), fermented pork (*Mam-Moo*), fermented pork sausage, fermented fish (*Pla-Som*) and fermented rice (*Khao Kab*), were collected from the markets in Bangkok, Thailand. One gram of each sample was enriched in a tube containing 10 mL lipolytic medium (0.3% (w/v) yeast extract, 0.3% (w/v) peptone, 0.01% (w/v) CaCl_2_ · 2H_2_O and 0.5% (v/v) Tween 20) supplemented with 200 mg·L^−1^ chloramphenicol (modified from Barrow and Feltham 1993) and incubated at 30°C for 3 days. After cultivation, the enrichment samples were streak on the lipolytic agar medium supplemented with 200 mg·L^−1^ chloramphenicol and the plates were incubated at 30°C for 3 days. Representative yeast colonies were picked up based on colonial characteristics and purified by single colony streaking method on yeast extract-malt extract (YM) agar medium [0.3% (w/v) yeast extract, 0.3% (w/v) malt extract, 0.5% (w/v) peptone, 1% (w/v) glucose and 1.5% (w/v) agar]. The yeast isolates were maintained on YM agar slant at 4°C, freeze in YM broth supplemented with 10% (v/v) glycerol at −80°C, and lyophilised for long-term preservation.

### Phenotypic characteristics

The morphological characteristics were performed as described by Yarrow (1998) and Kurtzman et al. (2011). For physiological characteristics, Yeast identification system API 20 C (bioMérieux, Marcy 1ʹ Etoile, France) was used according to the manufacturer’s instructions. The kit system allows the determination of the assimilation of 19 carbon sources for clinical isolates of pathogenic yeasts. The strips were incubated at 30°C for 48 h (24 to 48 h was recommended).

### 26S rDNA sequence and phylogenetic analysis

A loopful of yeast cells was suspended in 100 μL lysis buffer in a 1.5 mL microcentrifuge tube (Raeder and Broda 1985) and boiled in a water bath or metal heated block for 15 min. After boiling, 100 μL of 2.5 M potassium acetate (pH 7.5) was added, keep on ice for 2 h, and centrifuged at 14,000 rpm for 5 min. The upper layer was extracted twice with 100 μL chloroform/isoamyl alcohol [24:1 (v/v)] and DNA in the upper layer was precipitated with cold isopropanol, dried and dissolved in 30 μL MilliQ water. The D1/D2 domain of the large subunit ribosomal RNA gene (LSU rDNA D1/D2) was amplified using polymerase chain reaction (PCR) with primers F63 (5ʹ-GCATATCAATAAGCGGAGGAAAAG-3ʹ) and LR3 (5ʹ-GGTCCGTGTTTCAAGACGG-3ʹ) (O’Donnell 1993). The PCR condition was performed according to the methods of Kutzman and Robnett (1988). The PCR product was checked by agarose gel electrophoresis and purified using a GenepHlow^TM^ PCR clean up kit (Geneaid, New Taipei, Taiwan). The purified PCR product was sequenced at Macrogen (Korea). The sequences were compared with available sequence data using a BLASTN search on NCBI GenBank database (Altschul et al. 1997) and were aligned with sequences of related species retrieved from GenBank using the multiple alignment software CLUSTAL_X version 1.8 (Wanapat and Rowlinson 2007). The phylogenetic distances were calculated with the Kimura-2-parameter model (Kimura 1980) and tree topologies were inferred by using the neighbour-joining (Saitou and Nei 1987), which were constructed using MEGA 7.0 software (Kumar et al. 2016). The confidence values of the branch node were evaluated by using the bootstrap resampling method with 1,000 replications (Felsenstein 1985).

### Screening of lipolytic activity

Thirty-three yeast isolates were cultured on YM agar medium at 30°C for 48 h. After cultivation, each isolate was standardised the turbidity with McFarland standard solution No. 2 to obtain approximately 1 × 10^6^ CFU/mL in 0.85% NaCl and used as inoculum. One millilitres of the inoculum was added into 10 mL of the production medium containing 0.5% (w/v) yeast extract, 0.5% (w/v) peptone, 0.2% (w/v) glucose, 0.01% (w/v) MgSO_4_ · 7H_2_O, 0.1% (w/v) K_2_HPO_4_ and 1% (v/v) Tween 20 as a lipase inducer, pH 7.0, incubated by shaking (180 rpm) at 30°C for 72 h. The culture was centrifuged at 14,000 rpm for 10 min and the supernatant was analysed for lipolytic activity.

### Induction of lipolytic activity with palm and soy oils in liquid cultures

The seven selected isolates, 4E-1D, 5E-1T, 5E-2D, 8E-1T, 8E-1D, Fy-12 and Fy-13, were grown at 30°C for 48 h in 500 mL Erlenmeyer flask containing 100 mL YM broth. Each isolate was standardised the turbidity with McFarland standard solution No. 2 to obtain approximately 1 × 10^6^ CFU/mL in 0.85% NaCl and used as an inoculum. The inoculum (1%) was inoculated and cultivated in 100 mL of the production medium supplemented with 1% (v/v) of palm oil or soy oil under shaking condition at 30°C for 72 h. Cells were removed by centrifuging at 14,000 rpm for 10 min and the cultured supernatant were quantitatively determined for enzyme activity.

### Determination of lipolytic activity

The lipolytic activity was determined spectrophotometrically using *p*-nitrophenyl butyrate (*p*-NPB) as substrate (Fickers et al. 2003; Gomes et al. 2011). The substrate was prepared by dropwise addition of 0.1 mL of solution A (20.9 mg of *p*-NPB was dissolved in 10 mL absolute ethanol) into 10 mL of solution B (0.1 M sodium-potassium phosphate buffer, pH 7.2 containing 0.1 M NaCl) by using a volumetric flask, the final concentration of the substrate was 0.1 mM. The culture broth (20 μL) was added into 180 μL of the substrate and incubated for 60 minutes at 37°C. The release of *p*-nitrophenol was measured at 405 nm using a microplate reader (Wallac 1420, PERKINELMER, USA). The production medium was used as a control. One Unit of lipase activity was defined as the amount of enzyme releasing 1 μmole fatty acid per minute at the assay condition.

## Statistical analysis

All experiments were conducted in triplicate and statistical analysis was performed by SPSS 21.0 software (IBM, U.S.A). Analysis of variance was carried out using the ANOVA procedure by the Duncan method to determine significant (*p* ≤ 0.05) differences between the means.

## Results

**Figure 1.**
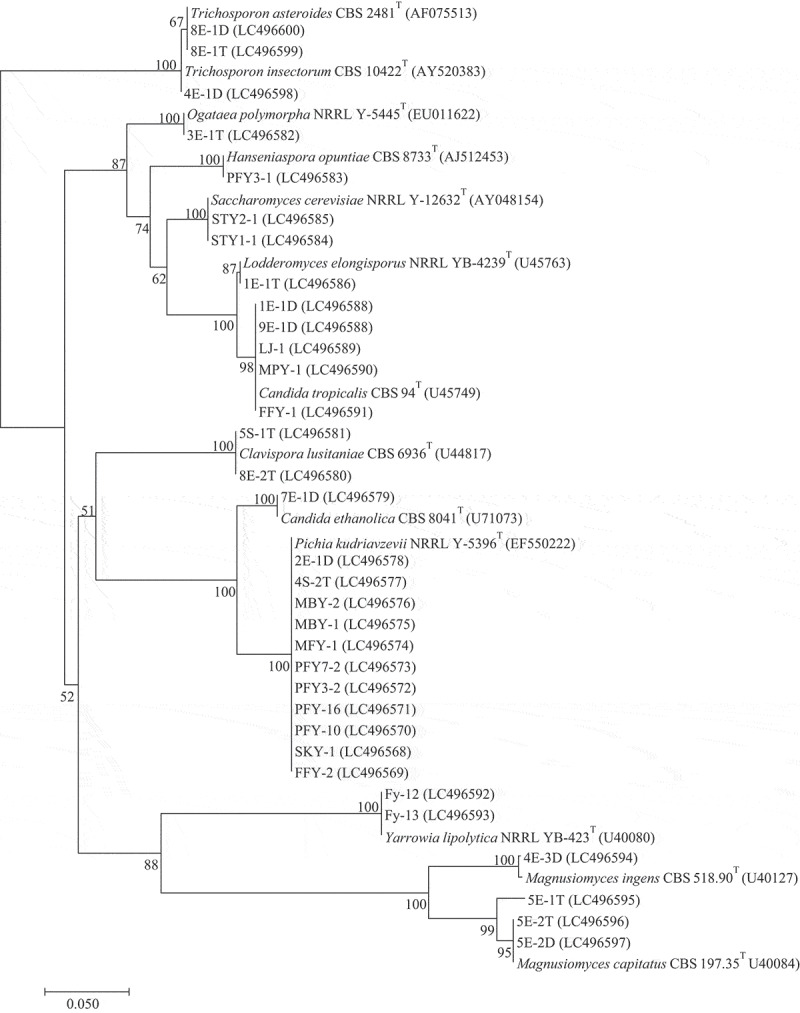
A neighbour-joining phylogenetic tree based on 26S rDNA gene sequences (LSU rDNA D1/D2) showing relationships between 33 isolates and the closely related type strains. The numbers at branch nodes indicate bootstrap percentages derived from 1000 replications (only value > 50% are shown at the node). Bar, 0.05 substitutions per nucleotide position

A total of 33 yeast isolates were characterised by the phenotypic characteristics based on the colony morphology and carbon utilisation. According to the taxonomic keys of Kurtzman et al. (2011), the isolated yeast genera were phenotypically identified as *Pichia, Candida, Magnusiomyces, Clavispora, Saccharomyces, Yarrowia, Trichosporon, Hanseniaspora, Lodderomyces* and *Ogataea*. Their differential colonial appearance and carbon utilisation such as adonitol, L-arabinose, xylitol, D-xylose, D-lactose, etc. are listed in Table 1. Accurately, the identification of the species level was done with the molecular method using the analysis of the D1/D2 domain of the 26S rRNA gene sequences (Table 2) and the neighbour-joining phylogenetic analysis (Figure 1).

**Table 1.** Differential characteristics of the isolates in Group I to XIII

Characteristics	I	II	III	IV	V	VI	VII	VIII	IX	X	XI	XII	XIII
Colony colour	Tannish-white	Cream	White	White	Cream	Cream	White	White	Tannish-white	Cream	White-cream	Cream	Cream
Carbon utilisation													
Adonitol	-	-	+	+	-	-	+	+	-	-	+	-	-
L-Arabinose	-	-	-	-	-	-	-	-	-	-	+	+	+
D-Cellobiose	-	-	+	+	+	+	-	+	-	-	+	+	+
D-Galactose	-	-	+	-	-	+	+	+	-	-	+	+	+
Glycerol	+	-	+	+	-	+	+	+	+	+	-	+	+
Calcium-2-keto-Gluconate	-	-	-	-	+	+	+	+	-	+	-	+	+
D-Glucose	+	+	+	+	+	+	+	+	+	+	+	+	+
α-methyl-D-Glucopyranoside	-	-	+	-	-	+	+	+	-	-	+	+	+
*N*-acetyl-Glucosamine	+	-	+	-	-	+	+	+	+	-	+	+	+
Inositol	-	-	-	-	-	-	-	-	-	-	-	-	-
D-Lactose	-	-	+	-	-	+	-	+	-	-	+	+	+
D-Maltose	-	-	+	+	-	+	+	+	-	-	+	+	+
D-Melezitose	-	-	+	+	-	-	+	+	-	-	+	+	+
D-Raffinose	-	-	+	-	-	+	-	-	-	-	+	-	-
D-Saccharose	-	-	+	+	-	+	+	+	-	-	+	+	+
D-Sorbitol	-	-	+	+	-	-	+	+	+	-	-	-	+
D-Trehalose	-	-	+	+	-	+	+	+	-	-	+	+	+
Xylitol	-	-	+	-	-	+	+	-	-	-	-	-	-
D-Xylose	-	-	+	+	-	-	+	+	-	-	-	+	+

Group I, *P. kudriavzevii* (11 isolates); Group II, *C. ethanolica* (1 isolate); Group III, *C. lusitaniae* (2 isolates); Group IV, *O. polymorpha* (1 isolate); Group V, *H. opuntiae*

(1 isolate); Group VI, *S. cerevisiae* (2 isolates); Group VII, *L. elongisporus* (1 isolate); Group VIII, *C. tropicalis* (3 isolates); Group IX, *Y. lipolytic* (2 isolates); Group X, *M. ingens* (1 isolate); Group XI, *M. capitatus* (3 isolates); Group XII, *T. insectorum* (1 isolate); Group XIII, *T. asteroides* (2 isolates). +, positive reaction; -, negative reaction

**Table 2.** Identification and lipolytic activity of yeasts isolated from foods and wastes

Group	Isolate no.	Isolation source	Similarity (%)*	Closest species	Lipase activity (U/mL)^†^
I	2E-1D	Palm cake	100	*P. kudriavzevii* NRRL Y-5396^T^	1.20 ± 0.05^b^
	4S-2T	Palm cake	100	*P. kudriavzevii* NRRL Y-5396^T^	1.86 ± 0.01^b,c,d^
	MBY-1	Fermented Beef	100	*P. kudriavzevii* NRRL Y-5396^T^	1.85 ± 0.02^b,c,d^
	MBY-2	Fermented Beef	100	*P. kudriavzevii* NRRL Y-5396^T^	2.38 ± 0.05^d,e^
	MFY-1	Fermented fish	100	*P. kudriavzevii* NRRL Y-5396^T^	2.36 ± 0.03^d,e^
	PFY3-2	Fermented fish	100	*P. kudriavzevii* NRRL Y-5396^T^	1.79 ± 0.05^b,c,d^
	PFY7-2	Fermented fish	99.9	*P. kudriavzevii* NRRL Y-5396^T^	1.89 ± 0.03^b,c,d^
	PFY-10	Fermented fish	100	*P. kudriavzevii* NRRL Y-5396^T^	1.05 ± 0.10^b^
	PFY-16	Fermented fish	100	*P. kudriavzevii* NRRL Y-5396^T^	1.05 ± 0.04^b^
	FFY-2	Fermented fish	100	*P. kudriavzevii* NRRL Y-5396^T^	1.11 ± 0.03^b^
	SKY-1	Fermented pork sausage	100	*P. kudriavzevii* NRRL Y-5396^T^	1.89 ± 0.05^b,c,d^
II	7E-1D	Palm fibre	100	*C. ethanolica* CBS 8041^T^	1.46 ± 0.68^b^
III	8E-2T	Palm cake	99.9	*Cl. lusitaniae* CBS 6936^T^	1.26 ± 0.04^b,c^
	5S-1T	Palm fibre	99.9	*Cl. lusitaniae* CBS 6936^T^	1.10 ± 0.04^b^
IV	3E-1T	Palm shell	100	*O. polymorpha* NRRL Y-5445^T^	1.16 ± 0.02^b^
V	PFY3-1	Fermented fish	100	*H. opuntiae* CBS 8733^T^	1.74 ± 0.07^b,c,d^
VI	STY1-1	Loog-Pang Khaomak	100	*S. cerevisiae* NRRL Y-12632^T^	1.81 ± 0.02^b,c,d^
	STY2-1	Loog-Pang Khaomak	100	*S. cerevisiae* NRRL Y-12632^T^	1.86 ± 0.03^b,c,d^
VII	1E-1T	Palm shell	100	*L. elongisporus* NRRL YB-4239^T^	0.17 ± 0.00^a^
VIII	1E-1D	Palm shell	99.8	*C. tropicalis* CBS 94^T^	1.86 ± 0.07^b,c,d^
	9E-1D	Palm fibre	100	*C. tropicalis* CBS 94^T^	2.20 ± 0.05^c,d,e^
	LJ-1	Fruits of *Myristica fragrans* Houtt.	100	*C. tropicalis* CBS 94^T^	3.12 ± 0.08^e^
	MPY-1	Fermented pork	100	*C. tropicalis* CBS 94^T^	1.99 ± 0.02^b,c,d^
	FFY-1	Fermented fish	100	*C. tropicalis* CBS 94^T^	1.92 ± 0.03^b,c,d^
IX	Fy-12	Fermented rice	99.9	*Y. lipolytica* NRRL YB-423^T^	5.99 ± 0.04^g^
	Fy-13	Fermented rice	100	*Y. lipolytica* NRRL YB-423^T^	5.21 ± 0.09^f^
X	4E-3D	Palm shell	99.7	*M. ingens* CBS 518.90^T^	1.21 ± 0.06^b^
XI	5E-1T	Palm shell	98.3	*M. capitatus* CBS 197.35^T^	7.77 ± 0.32^i^
	5E-2T	Palm shell	100	*M. capitatus* CBS 197.35^T^	1.16 ± 0.07^b^
	5E-2D	Palm shell	100	*M. capitatus* CBS 197.35^T^	5.26 ± 0.17^e,f^
XII	4E-1D	Palm shell	100	*T. insectorum* CBS 10422^T^	45.68 ± 2.37^j^
XIII	8E-1T	Palm cake	100	*T. asteroides* CBS 2481^T^	7.10 ± 0.08^h^
	8E-1D	Palm cake	100	*T. asteroides* CBS 2481^T^	5.96 ± 0.13^f^

*Sequence similarity with the type strain of the species concerned in the D1/D2 domain of the 26S rRNA gene

^†^Mean values with the same letter do not statistically differ from each other by the ANOVA Duncan test (*p* = 0.05)

We found 11 species in the ascomycetes which were identified as *Pichia kudriavzevii* (Group I: SKY-1, FFY-2, PFY-10, PFY-16, PFY3-2, PFY7-2, MFY-1, MBY-1, MBY-2, 4S-2T, and 2E-1D); *Candida ethanolica* (Group II, 7E-1D); *Clavispora lusitaniae* (Group III, 8E-2T and 5S-1T); *Ogataea polymorpha* (Group IV, 3E-1T); *Hanseniaspora opuntiae* (Group V, PFY3-1); *Saccharomyces cerevisiae* (Group VI, STY1-1 and STY2-1.); *Lodderomyces elongisporus* (Group VII, 1E-1T); *Candida tropicalis* (Group VIII, 1E-1D, 9E-1D, LJ-1, MPY-1 and FFY-1); *Yarrowia lipolytica* (Group IX, Fy-12 and Fy-13); *Magnusiomyces ingens* (Group X, 4E-3D) and *Magnusiomyces capitatus* (Group XI, 5E-1 T, 5E-2T and 5E-2D). Two species in the basidiomycetes containing the isolate 4E-1D (Group XII) which was identified as *Trichosporon insectorum* and 2 isolates of *Trichosporon asteroides* (Group XIII, 8E-1T and 8E-1D).

The isolation rate of lipolytic yeasts in this study varied among different sources including foods, fruits, and wastes of palm oil factory which was founded in *P. kudriavzevii* (33.4%), *C. tropicalis* (15.2%), *M. capitatus* (9.1%), *Cl. lusitaniae* (6.1%), *S. cerevisiae* (6.1%), *Y. lipolytica* (6.1%), *T. asteroides* (6.1%), *C. ethanolica* (3.0%), *M. ingens* (3.0%), *H. opuntiae* (3.0%), *L. elongisporus* (3.0%), *O. polymorpha* (3.0%) and *T. insectorum* (3.0%).

**Figure 2.**
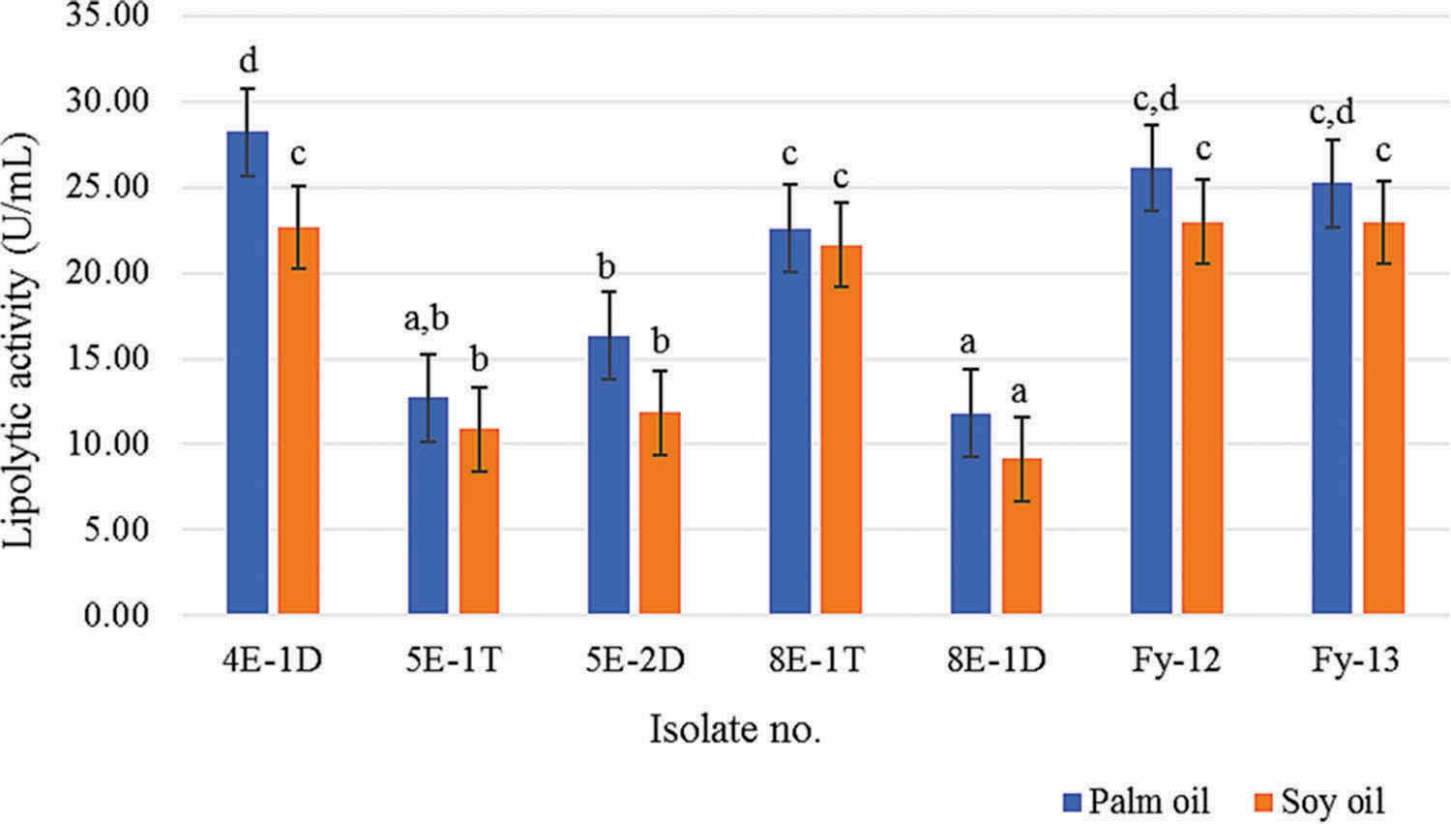
Lipolytic activity of the seven selected isolates, 4E-1D, 5E-1T, 5E-2D, 8E-1T, 8E-1D, Fy-12 and Fy-13 after cultivation in the medium containing palm oil and soy oil as carbon sources for 72 h. Mean values with the same letter do not statistically differ from each other by the ANOVA Duncan test (*p* = 0.05)

The quantitative screening on Tween 20 liquid culture revealed varying levels of lipase activity. Most isolates showed lipolytic activity ranging from 1.05 to 3.12 U/mL (Table 1). Isolate 4E-1D exhibited the highest activity of 45.68 ± 2.37 followed by the isolate 5E-1T, 5E-2D, 8E-1T, 8E-1D, Fy-12 and Fy-13 with the lipase activities of 7.77 ± 0.32, 5.26 ± 0.17, 7.10 ± 0.08, 5.96 ± 0.13, 5.99 ± 0.04 and 5.21 ± 0.09 U/mL, respectively, while the isolate 1E-1T showed the lowest lipase activity of 0.17 ± 0.00 U/mL. The seven best lipolytic strains, 4E-1D, 5E-1T, 5E-2D, 8E-1T, 8E-1D, Fy-12 and Fy-13, were chosen and further screened using palm oil and soy oil as the carbon sources because they are low-cost materials for enzyme production. As shown in Figure 2, the best lipase producers in both palm oil and soy oil were *T. insectorum* strain 4E-1D (28.19 ± 4.84 U/mL in palm oil and 22.63 ± 0.18 U/mL in soy oil), *Y. lipolytica* strain Fy-12 (26.11 ± 2.10 and 22.97 ± 0.69 U/mL in palm and soy oils, respectively) and *Y. lipolytica* strain Fy-13 (25.20 ± 1.86 U/mL in palm oil and 22.95 ± 0.24 U/mL in soy oil). The other selected isolates, 5E-1T, 5E-2D, 8E-1T and 8E-1D, also exhibited lipolytic activity varying from 11.79 to 16.33 U/mL in palm oil and from 9.14 to 11.84 U/mL in soy oil.

## Discussion

Currently, *Candida tropicalis, Diutina rugosa, Moesziomyces antarcticus, Candida cylindracea, Candida parapsilosis, Cutaneotrichosporon curvatum, Yarrowia deformans, Rhodotorula glutinis, Rhodotorula pilimanae, Yarrowia lipolytica, Pichia bispora, Pichia xylosa, Pichia mexicana, Pichia silvicola, Pichia burtonii, Saccharomyces cerevisiae* and *Trichosporon asteroides* were found to be the predominant terrestrial lipase-producing yeasts (Vakhlu and Kour 2006; Ciafaidini et al. 2006; Treichel et al. 2010). Thus, *C. ethanolica* strain 7E-1D, *M. ingens* strain 4E-3D, *M. capitatus* isolates 5E-1T, 5E-2T and 5E-2D, *H. opuntiae* strain PFY3-1, *Lodderomyces elongisporus* isolate 1E-1T, *Ogataea polymorpha* isolate 3E-1T, *Pichia kudriavzevii* strains SKY-1, FFY-2, PFY-10, PFY-16, PFY3-2, PFY7-2, MFY-1, MBY-1, MBY-2, 4S-2T, and 2E-1D, and *Trichosporon insectorum* strain 4E-1D obtained in this study could be assumed as the new lipolytic yeasts. Moreover, this is the first report of lipolytic yeasts isolated from wastes of the palm oil industry and fermented foods in Thailand.

According to our results, three strains of the genus *Trichosporon, T. insectorum* 4E-1D, *T. asteroides* 8E-1T and *T. asteroides* 8E-1D, measured in this study exhibited high lipolytic activity. These findings were correlated with previous studies which indicated some species of the genus *Trichosporon* produced the high level of lipase activity. Chen et al. (1992) reported that *T. fermentans* strain WU-C12 isolated from soil displayed high lipase production of 128 U/mL. Santos et al. (2007) also found that *Trichosporon* spp. obtained from crude cheese produced 7.3 U/mL of lipase after cultivation for 72 h. Furthermore, it has been reported that *T. asahii* strain MSR 54, isolated from petroleum sludges, produced 104 U/mL of lipase in the liquid medium containing corn oil as a carbon source (Kumar and Gupta 2008).

In addition, the two isolates of the genus *Magnusiomyces, M. capitatus* strains 5E-1T and 5E-2D, and the two strains of the genus *Yarrowia, Y. lipolytica* strains Fy-12 and Fy-13 also showed significant lipolytic activity. These results were in accordance with several studies as described previously. Salgado et al. (2019) revealed that *M. capitatus* strain JT5 isolated from olive mill wastewaters exhibited 3.96 U/mL of lipase activity after cultured in the production medium containing olive oil as a carbon source. *Y. lipolytic* was well-known as the lipase-producing yeast. Corzo and Revah (1999) had studied and found that *Y. lipolytica* strain 681 produced 31.9 and 30.9 U/mL in the production medium supplemented with corn oil and olive oil, respectively. Additionally, Domínguez et al. (2003) reported that the high lipolytic activity level of *Y. lipolytica* strain CECT 1240 was obtained with the liquid culture containing sunflower oil (58 U/mL), olive oil (49 U/mL) and tributyrin (33 U/mL). This study suggested that *Trichosporon, Magnusiomyces* and *Yarrowia* strains could be further studied and used for lipase production.

## Conclusion

The present study showed that Thai fermented foods and the palm oil industrial wastes could be employed as a valuable source for the isolation of lipolytic yeasts. Thirty-three isolates can be divided into 2 groups including ascomycetous (30 isolates) and basidiomycetous (3 isolates) yeasts. The seven isolates, *Trichosporon insectorum* 4E-1D, *Magnusiomyces capitatus* 5E-1T and 5E-2D, *Trichosporon asteroides* 8E-1T and 8E-1D, and *Yarrowia lipolytica* Fy-12 and Fy-13, exhibited a significant level of lipolytic activity in Tween 20 liquid culture and were chosen for studying the lipolytic activity in palm and soy oils. Each selected strain showed varying levels of lipolytic activity in both palm and soy oils ranging from 9.14 to 28.19 U/mL. The further study focuses on the optimisation of lipase production by the best lipase producer, *T. insectorum* strain 4E-1D, as well as the characterisation and purification of lipase from the liquid medium which may provide for the commercial exploitation.
